# Integrin α_v_β_3_-targeted radionuclide therapy combined with immune checkpoint blockade immunotherapy synergistically enhances anti-tumor efficacy

**DOI:** 10.7150/thno.39203

**Published:** 2019-10-16

**Authors:** Haojun Chen, Liang Zhao, Kaili Fu, Qiuming Lin, Xuejun Wen, Orit Jacobson, Long Sun, Hua Wu, Xianzhong Zhang, Zhide Guo, Qin Lin, Xiaoyuan Chen

**Affiliations:** 1 Department of Nuclear Medicine & Minnan PET Center, Xiamen Cancer Hospital, The First Affiliated Hospital of Xiamen University, China;; 2 Department of Radiation Oncology, Xiamen Cancer Hospital, The First Affiliated Hospital of Xiamen University Teaching Hospital of Fujian Medical University, Xiamen, China;; 3 State Key Laboratory of Molecular Vaccinology and Molecular Diagnostics & Center for Molecular Imaging and Translational Medicine, School of Public Health, Xiamen University, Xiamen, China;; 4 Laboratory of Molecular Imaging and Nanomedicine, National Institute of Biomedical Imaging and Bioengineering, National Institutes of Health, Bethesda, Maryland, USA.

**Keywords:** Targeted radionuclide therapy, immunotherapy, programed death ligand 1 (PD-L1), ^177^Lu-EB-RGD, combination treatment

## Abstract

Rationale: Radiotherapy combined with immunotherapy has revealed promising outcomes in both preclinical studies and ongoing clinical trials. Targeted radionuclide therapy (TRT) is a branch of radiotherapy concerned with the use of radioisotopes, radiolabeled molecules or nanoparticles that deliver particulate radiation to cancer cells. TRT is a promising approach in cases of metastatic disease where conventional treatments are no longer effective. The increasing use of TRT raises the question of how to best integrate TRT with immunotherapy. In this study, we proposed a novel therapeutic regimen that combined programmed death ligand 1 (PD-L1)-based immunotherapy with peptide-based TRT (^177^Lu as the radionuclide) in the murine colon cancer model.

Methods: To explore the most appropriate timing of immunotherapy after radionuclide therapy, the anti-PD-L1 antibody (αPD-L1 mAb) was delivered in a concurrent or sequential manner when ^177^Lu TRT was given.

Results: The results demonstrated that TRT led to an acute increase in PD-L1 expression on T cells, and TRT in combination with αPD-L1 mAb stimulated the infiltration of CD8^+^ T cells, which improved local tumor control, overall survival and protection against tumor rechallenge. Moreover, our data revealed that the time window for this combination therapy may be critical to outcome.

Conclusions: This therapeutic combination may be a promising approach to treating metastatic tumors in which TRT can be used. Clinical translation of the result would suggest that concurrent rather than sequential blockade of the PD-1/PD-L1 axis combined with TRT improves overall survival and long-term tumor control.

## Introduction

With the recent success of cancer immunotherapy, especially immune checkpoint blockade (ICB) therapy, there has been renewed interest in this treatment modality 1. Several kinds of ICB monoclonal antibodies (mAbs) have been applied in preclinical models of cancer and in patients, targeting cytotoxic T-lymphocyte antigen 4 (CTLA-4) 2, programmed death 1 (PD-1), PD-1 ligand 1 (PD-L1) 3, transforming growth factor (TGF)-β 4, blocking immune checkpoints, and facilitating anti-tumor activity. Research has shown that PD-L1, which can inhibit CD8^+^ T-cell effector function by interacting with PD-1, is expressed on many types of tumor cells, tumor-associated macrophages (TAMs), and other cells in the tumor immune microenvironment 5. Therefore, ICB using anti-PD1/PD-L1 mAbs has emerged as a promising treatment strategy in various types of malignancies. Indeed, the PD-1/PD-L1 mAbs pembrolizumab, nivolumab, and atezolizumab have been approved by the United States Food and Drug Administration (FDA) to treat patients with advanced non-small-cell lung carcinoma (NSCLC), head and neck squamous cell carcinoma (HNSCC), and melanoma 6-8.

However, only 15-40% of patients respond to anti-PD1/PD-L1 mAbs; therefore, the population of patients benefiting from ICB therapy is still rather narrow 1. As such, researchers must elucidate how to combine ICB therapy with other treatments. Recently, numerous studies have confirmed that systemic anti-tumor immunity is augmented following radiotherapy combined with ICB therapy targeting PD-1/PD-L1 in preclinical models of melanoma, breast cancer, lung cancer and colon cancer. 4, 9, 10. These studies suggest that radiotherapy can induce a local inflammatory response and simultaneously upregulate PD-L1 expression in the tumor microenvironment, which gives an opportunity to use anti-PD-L1 therapy following radiotherapy for enhancing the therapeutic benefits of immunotherapy in cancer 11. Moreover, a range of preclinical models have suggested that irradiated tumor cells can act as an “*in situ* vaccine”, releasing tumor immunogenic antigen, enhancing immunogenicity, and inducing a systemic anti-tumor immune response outside of the irradiated tumor field 12, 13. This has been termed the “abscopal effect” and may be further strengthened in the presence of immunotherapy 12, 14. More recently, phase III PACIFIC clinical trial has demonstrated that the αPD-L1 mAb (Durvalumab) combined with concurrent chemoradiotherapy could extend overall survival than standard chemoradiotherapy 15. Taken together, the combination of radiotherapy with PD1/PD-L1 mAbs shows improved anti-tumor efficacy, providing a rationale for this combined therapeutic strategy.

Targeted radionuclide therapy (TRT) is a branch of radiotherapy that uses radionuclides, radiolabeled molecules, or nanoparticles that either naturally accumulate in or are designed to target tumors. Unlike external-beam radiotherapy, which mostly acts locally on primary tumors, TRT can also be used to treat metastatic tumors because it can be administered systemically 16. The major difference between the two forms of radiotherapy is dose rate. External-beam radiotherapy is delivered at high dose rates, typically about 6 Gy/min in short, repeated daily fractions of about 2 Gy. In TRT, however, the absorbed dose is delivered continuously but slowly, with a dose rate of 0.01-1.00 Gy/h that tapers over time as the radionuclide decays 16. Another difference lies in the absorbed dose. Regarding TRT, a high inter-patient variability of the mean absorbed doses to lesions was found that could be explained by variable receptor densities between individuals and different types of targeting vectors. In general, it should be noted that on average the absorbed dose of TRT was less than those achieved with external beam radiotherapy. For example, the mean absorbed doses in tumor lesions for ^177^Lu-PSMA-617, ^177^Lu-DOTA-TATE and ^177^Lu-DOTA-TOC are 13.1, 9.7 and 7.5 Gy/GBq, respectively 17, 18. Differing dose rates and absorbed doses have been shown to cause differences in gene expression, mode of cell death, and type of DNA damage that is induced 19, 20. However, the radiobiological effects of the two forms of radiotherapy must be better understood. Peptide-radiometal conjugates that bind to cell surface somatostatin receptors have been used clinically for many years in an approach known as peptide receptor radionuclide therapy (PRRT). On January 26, 2018, the FDA approved ^177^Lu-labeled dodecanetetraacetic acid tyrosine-3-octreotate (DOTA) to treat somatostatin receptor-positive gastroenteropancreatic neuroendocrine tumors 21. More recently, ^177^Lu-labeled prostate specific membrane antigen ligand has been tested in metastatic castration-resistant prostate cancer, with promising preliminary results 22, 23.

Since there is evidence from preclinical and clinical studies to suggest that external-beam radiotherapy boosts the therapeutic benefits of immunotherapy, researchers must now elucidate how to best combine TRT with immunotherapy. To this end, they must design therapeutic protocols that combine the strengths of TRT with those of immunotherapy, taking into consideration optimal timing and sequencing. In the present paper, we proposed a novel therapeutic regimen combining PD-L1-based immunotherapy with PD-L1 antibody treatment with peptide-based TRT. The peptide containing Arg-Gly-Asp (RGD) sequence specifically targets the cell surface receptor integrin α_v_ß_3_, which is overexpressed in various malignancies 24. As such, RGD was identified as a promising radionuclide vector and was modified for TRT application in the present study. To further enhance the efficacy of TRT, the RGD peptide was chemically conjugated with an albumin-binding moiety—truncated Evans Blue (denoted as EB-RGD). In our previous study, EB-RGD showed significantly higher tumor uptake and tumor residence time than RGD monomer 25. In the present study, EB-RGD was labeled with the β-emitting radionuclide ^177^Lu for TRT purposes. Furthermore, to evaluate the most appropriate timing of immunotherapy after radiotherapy, the anti-PD-L1 mAb was delivered in a concurrent or sequential manner when ^177^Lu TRT was given. Through this investigation, we aimed to explore whether this combined therapeutic regimen would synergistically enhance anti-tumor efficacy and to propose a rationale for the combination of TRT and immunotherapy.

## Materials and methods

### General

All chemicals were purchased from Sigma-Aldrich Chemical Co. or Thermo Fisher Scientific. EB-NH_2_ was synthesized as described previously 26, 27. Cyclic-(Arg-Gly-Asp-D-Phe-Lys) (RGD) peptide was purchased from C.S. Bio. InvivoPlus anti-mouse PD-L1 mAb was purchased from BioXCell (Cat. No. BP0101). The murine colon adenocarcinoma cell line, MC38, was purchased from the China National Infrastructure of Cell Line Resource. Labeling efficiency and radiochemical purity were tested using a Mini-Scan radio-TLC Scanner (BioScan, USA) and Dionex Ulti-Mate 3000 high performance liquid chromatography (HPLC; Thermo Scientific, USA), with a flow-counter radioactivity detector (BioScan, USA). Radioactivity was measured using a γ-counter (WIZARD 2480; Perkin-Elmer, USA) and CRC-25R Dose Calibrators (CAPIN-TEC Inc., USA). SPECT imaging was performed using a nanoScan SPECT/CT scanner (Mediso, Hungary).

### Chemistry and radiochemistry

EB-RGD was synthesized as in our previous study 25. Briefly, EB-RGD was prepared by linking truncated Evans Blue molecules to a lysine spacer, attaching a chelator to the α-amine, attaching maleimide to the ε-amine, and attaching the thiolated RGD peptide to the maleimide. DOTA was used as the chelator to allow complexation with the radionuclide ^177^Lu.

High-purity lutetium chloride was obtained from itG (Germany) as a sterile solution of ^177^LuCl_3_ in 0.05 M HCl, with a radionuclide purity of > 99%. For radiolabeling, either 1.85 GBq (50 mCi, for EB-RGD) or 0.55 GBq (15mCi, for DOTA-RGD) of ^177^LuCl_3_ was diluted using 0.3 mL of 0.5 M NH_4_OAc (pH = 5.6); EB-RGD (50 μg dissolved in 10 μL absolute ethyl alcohol) or RGD (50 μg dissolved in 100 μL metal-free water) was then added. The mixture was heated for 30 min at 90 °C, purified using a C_18_ cartridge, and passed through a 0.22-μm aseptic filtration membrane. Radiochemical purity control was performed using analytical thin-layer chromatography (Bioscan, USA). CH_3_OH:NH_4_OAc (v/v 1:1) was used as the developing solution.

### Preparation of tumor-bearing mice

Seven- to 8-week-old female C57BL/6 mice from Beijing Vital River Laboratory Animal Technology Co., Ltd. were used in the present study. All animal studies were approved by the Animal Care and Use Committee of the First Affiliated Hospital of Xiamen University. The MC38 murine colon adenocarcinoma was selected as the tumor model in this study. This tumor model is generally PD-L1 positive expression and is used as an immunoresponsive murine model in many immunological researches 28. Besides, previous study has confirmed that this tumor model has a positive expression of integrin α_v_ß_3_ 29. To induce MC38 tumors, 10^6^ cells in PBS (pH=7.4) were injected subcutaneously into the right rear flank of each mouse. The mice then received either saline, anti-PD-L1 mAb, ^177^Lu-EB-RGD, or combined therapy when the tumor volume reached about 100 mm^3^ (7 days after inoculation) and underwent small animal SPECT imaging studies when the tumor volume reached 200 mm^3^ (10-12 days after inoculation).

### SPECT imaging and biodistribution study

SPECT was performed using a four-head multiplexing multipin hole camera (Mediso, Hungary). Healthy MC38 tumor-bearing female C57BL/6 mice were intravenously injected with 17-18 MBq of either ^177^Lu-EB-RGD (n = 4) or ^177^Lu-RGD (n = 4). Whole body SPECT images were acquired at 4, 24, 48, 72, and 96 hours post injection (p.i.). SPECT images were obtained with a time-per-view of 30-60 seconds, resulting in a scan time of 25-60 min (window width: 20%, matrix: 256 × 256, medium zoom). After acquisition, SPECT data were reconstructed iteratively with a software (Tera-Tomo^TM^) using ^177^Lu γ-energies of 56.1, 112.9, and 208.4 keV. The SPECT images were analyzed using InVivoScope software (Bioscan Inc.). To quantify the radioactive signals in mouse tumors and other tissues, regions of interest (ROIs) were drawn and counted on the SPECT images of tumor, liver, heart, and muscle. The results were expressed as tumor-to-muscle (T/M), liver-to-muscle (L/M), and blood-to-muscle (B/M) ratios.

For the biodistribution study, C57BL/6 mice bearing MC38 tumors and injected with ^177^Lu-EB-RGD (0.5 MBq) were sacrificed at 4, 24, 48, and 72 h p.i. Blood, muscle, bone, liver, kidneys, spleen, intestine, heart, lung, muscle, brain, stomach, and tumor were collected and wet weighed. The biodistribution of ^177^Lu-RGD was also measured for comparison. Mice bearing MC38 tumors were sacrificed at 4 h p.i. and their main organs were collected and weighed. Radioactivity was assayed using a γ-counter, and the results were expressed as percentage of injected dose per gram (%ID/g).

### *In vivo* therapy regimen

**Figure 1:**
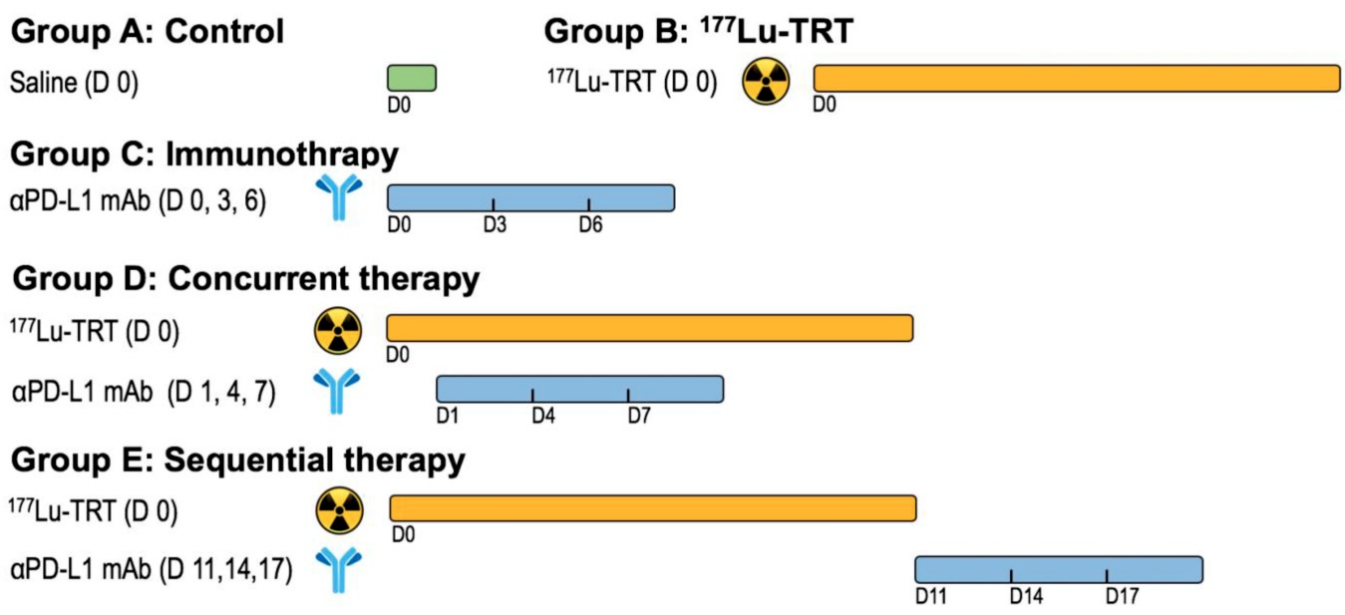
Design of therapy protocol. Group A received saline. Group B received 18.5 MBq of ^177^Lu-EB-RGD targeted radionuclide therapy (TRT) by intravenous injection. Group C received anti-PD-L1 mAbs (αPD-L1 mAb) immunotherapy at a dose of 10mg/kg by intraperitoneal injection (d 0, 3 and 6). Group D received concurrent combined therapy with ^177^Lu-EB-RGD (d 0) and αPD-L1 mAb (d 1, 4 and 7), and group E received sequential combined therapy with ^177^Lu-EB-RGD (d 0) and αPD-L1 mAb (d 11, 14 and 17).

One week after inoculation with MC38 tumor cells, C57BL/6 mice were divided into five treatment groups of 9 to 10 mice per group: Group A received saline, Group B, 18.5 MBq of ^177^Lu-EB-RGD by intravenous (i.v.) injection, Group C, anti-PD-L1 mAb on days 0, 3, and 6 at a dose of 10 mg/kg by intraperitoneal (i.p.) injection, Group D received concurrent combined therapy, with 18.5 MBq of i.v. ^177^Lu-EB-RGD given on day 0 and 10 mg/kg i.p. of anti-PD-L1 mAb given on days 1, 4 and 7, and Group E received sequential combined therapy, with 18.5 MBq of i.v. ^177^Lu-EB-RGD given on day 0 and 10 mg/kg of i.p. anti-PD-L1 mAb given on days 11, 14, and 17. The overall therapy regimen is shown in Figure 1.

To evaluate the anti-tumor efficacy of the treatment, tumor volume and body weight were monitored every 2 days. Individual tumor size was calculated using the following formula: length × width × width)/2. Groups A, B, C, and D underwent ^18^F-FDG positron emission tomography (PET) imaging 3 days after the start of treatment. The PET imaging protocol and analysis were performed as previously described 30, except that the animals were fasted for 4 h before ^18^F-FDG PET.

The endpoint criteria for Kaplan-Meier analysis were as follows: weight loss of > 15%, tumor volume of > 1,500 mm^3^, active tumor ulceration, and abnormal behavior indicating pain or unease.

### Flow cytometry

The mouse tumor tissue was obtained and the necrotic tissues and fat were removed. The tumor tissue was cut into small pieces (about 2 mm) and placed in Dulbecco's modified Eagle's medium with 1 mg/mL collagenase IV (Sigma-Aldrich, Cat. No. C5138) and 0.1 mg/mL DNase I (Sigma-Aldrich Cat. No. D4527) for 30-40 minutes at 37 ºC. The cell suspension was then filtered through a cell strainer, washed with cell staining buffer (BD Pharmingen^TM^; Cat. No. 554656), and centrifuged for 7 min at 300 × g. The supernatant was then discarded.

Viable cells were counted and resuspended in cell staining buffer at a concentration of 5-10 × 10^6^ cells/mL. Next, 100 µL/tube of cell suspension (5-10 x 10^5^ cells/tube) was distributed into 2-mL plastic tubes. Fc receptors were then blocked by pre-incubation in 1 µg of purified anti-mouse CD16/CD32 mAb (BD Pharmingen^TM^, Cat. No. 553141) per 10^6^ cells in 100 µL at 4 ºC for 5 min. Then, 100 μL of cells per tube were incubated with fluorescent mAbs specific to mouse CD45, CD11b, CD274, CD3e, or CD8α (BD Pharmingen^TM^; Cat. Nos. 553018, 557396, 564715, 553061, and 553032, respectively) for 30 min at 2 -8 °C. The tubes were then washed once in 2 mL of stain buffer and centrifuged at 350 × g for 5 min. According to the intracellular staining protocol for FoxP3 staining (BD Pharmingen^TM^; Cat. No. 563101), the cells were analyzed and data acquired using the ACEA NovoCyte Flow Cytometer.

### Multiplexed immunofluorescence

Multiplexed immunofluorescence (IF) was performed by sequentially staining 4-µm-thick formalin-fixed, paraffin-embedded whole tissue sections with standard, primary mAbs paired with a unique fluorochrome, followed by staining with DAPI. For example, deparaffinized slides were incubated with anti-CD8 mAb (#CST98941; Cell Signaling Technology) for 30 min and then treated with anti-rabbit horseradish peroxidase (HRP)-conjugated secondary mAb (PV-6001; Zhongshan Bio) for 10 min. IF labeling was then developed for 10 min using Alexa Fluor™ 488-labeled tyramide (Thermo Fisher; B40953), as per the manufacturer's instructions. The slides were then washed in Tris buffer for 5 min, transferred into preheated citrate solution (90 °C), and heat-treated using a microwave set at 20% maximum power for 15 min. The slides were cooled in the same solution to room temperature (RT). Between all steps, the slides were washed in Tris buffer. The same process was repeated for the following mAbs and fluorescent dyes, in order: Anti-CD11b mAb (Abcam, ab133357)/Alexa Fluor™ 647-labeled tyramide, CD45 rabbit mAb (Cell Signaling Technology)/Alexa Fluor™ 488-labeled tyramide, CD274 mAb (Absin; abs136046)/Alexa Fluor™ 594-labeled tyramide, CD8α mAb (Cell Signaling Technology; 98941)/Alexa Fluor™ 546-labeled tyramide. Each slide was then treated with two drops of DAPI (D1306; Thermo Fisher), washed in distilled water, and manually cover-slipped. The slides were then air-dried and mounted with Prolong Diamond Anti-fade mounting medium (P36965; Thermo Fisher). Pictures were taken with the Aperio Versa 8 tissue imaging system (Leica). Images was analyzed using Indica Halo software.

### Histopathological staining after therapy

MC38 tumor samples from groups A-D were collected and sectioned at day 7 after therapy, and samples from group E were collected at day 14 after therapy. To visualize the endothelial cells, CD31 immunohistochemistry was performed. In brief, tumor sections were fixed using cold acetone and rinsed in PBS; endogenous peroxidase activity was blocked with a 3% H_2_O_2_ aqueous solution for 10 mins and unspecific binding of proteins was blocked by using 1% bovine serum albumin solution for 1 h at RT. The slides were incubated with a 1:100 dilution of anti-mouse CD31 (Abcam, ab9498) mAb at RT overnight and then incubated with peroxidase-conjugated rabbit-anti-mouse secondary mAb. Next, 3,3'-diaminobenzidine (DAB) was used to visualize peroxidase activity in the sections. For Ki-67 staining, tumor sections were fixed with cold acetone for 20 min and then air-dried for 30 min at RT. Blocking was achieved using 1% bovine serum albumin for 30 min, and the slides were stained with Ki-67-specific mAb (Abcam; ab15580) then incubated with peroxidase-conjugated rabbit-anti-mouse secondary mAb. DAB was then used to visualize peroxidase activity in the sections. Immunofluorescent terminal deoxynucleotidyl transferase-mediated dUTP-biotin nick-end labeling (TUNEL) analysis was carried out using a commercially available kit (Roche Applied Science). In accordance with the manufacturer's specifications, samples were fixed using 10% formalin and permeabilized in 0.1% Triton for 2 min on ice. Next, 50 mL of TUNEL reaction mixture were added to the samples. The slides were incubated in a humidified atmosphere for 60 min at 37 ºC in the dark. After rinsing in PBS and mounting in a DAPI-containing medium, the samples were observed under an epifluorescence microscope in the green fluorescent protein channel. Hematoxylin and eosin staining of mouse tissues was conducted by Univ-Bio Inc.

### Statistics

Quantitative data were expressed as mean ± SD. Means were compared using one-way analysis of variance and Student's *t* test. *P* value of < 0.05 was considered statistically significant.

## Results

### Preparation of radiolabeled compounds

In the present study, we prepared two radioligands: ^177^Lu-EB-RGD, which has high affinity for integrin and contains an albumin binding moiety, and ^177^Lu-RGD, which has high affinity for the integrin receptor but lacks the albumin-binding moiety. The ^177^Lu-EB-RGD and ^177^Lu-RGD were radiolabeled at an average specific activity of 55.85 ± 14.0 and 18.6 ± 4.7 GBq/μmol, respectively, with greater than 95% radiochemical purity after purification.

### SPECT imaging and biodistribution of ^177^Lu-EB-RGD in MC38 tumor-bearing mice

**Figure 2:**
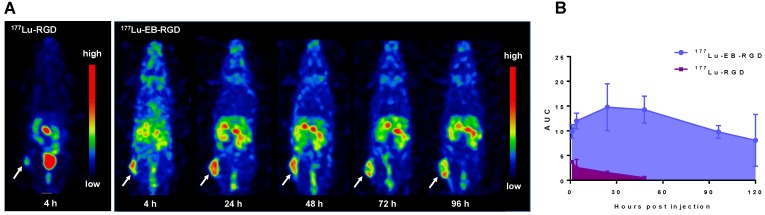
(A) Projection SPECT images of MC38 tumor bearing mice at 4, 24, 48, 72 and 96 h after injection of 18.5 MBq ^177^Lu-EB-RGD (n = 4). (B) Projection SPECT images of MC38 tumor bearing mice at 1 and 4 h after injection of 18.5 MBq ^177^Lu-RGD (n = 4). (C) Area under the curves (AUC) of ^177^Lu-EB-RGD (blue) and ^177^Lu-RGD (purple) calculated from the radioactivity accumulation in the tumor over time. Arrows indicate tumor location.

To investigate the *in vivo* behavior of ^177^Lu-EB-RGD, we performed small-animal SPECT imaging studies. Representative whole-body SPECT images of C57BL/6 mice bearing MC38 tumors are shown in Figure 2. In the case of ^177^Lu-EB-RGD, high tumor-to-background ratio (T/M: 9.03 ± 0.44) was observed 4 h p.i., with the tumor uptake peaking at 24 h p.i. (T/M: 14.87 ± 0.88) and reduced slightly over time. Meanwhile, ^177^Lu-EB-RGD uptake in the blood was relatively high at 1 h p.i. (B/M: 5.22 ± 0.23) but decreased gradually from 24 to 96 h p.i. The ^177^Lu signal was also observed in the liver and kidneys, which also decreased from 24 to 96 h p.i. The clearance of ^177^Lu-EB-RGD from non-target tissues allowed the tumor isografts to be visualized (Figure 2A). SPECT imaging with ^177^Lu-RGD was also performed in MC38 tumor-bearing mice (Figure 2B). Compared with ^177^Lu-EB-RGD, ^177^Lu-RGD was rapidly cleared from the blood through the urinary tract and showed significantly lower accumulation in tumors (T/M: 2.90 ± 0.30) at 4 h p.i. The encouraging high tumor uptake of ^177^Lu-EB-RGD, coupled with prolonged tumor retention time resulted in high exposure to the radioactive isotope, as evidenced from area under the curve calculated from non-decay corrected data (Figure 2C).

**Figure 3:**
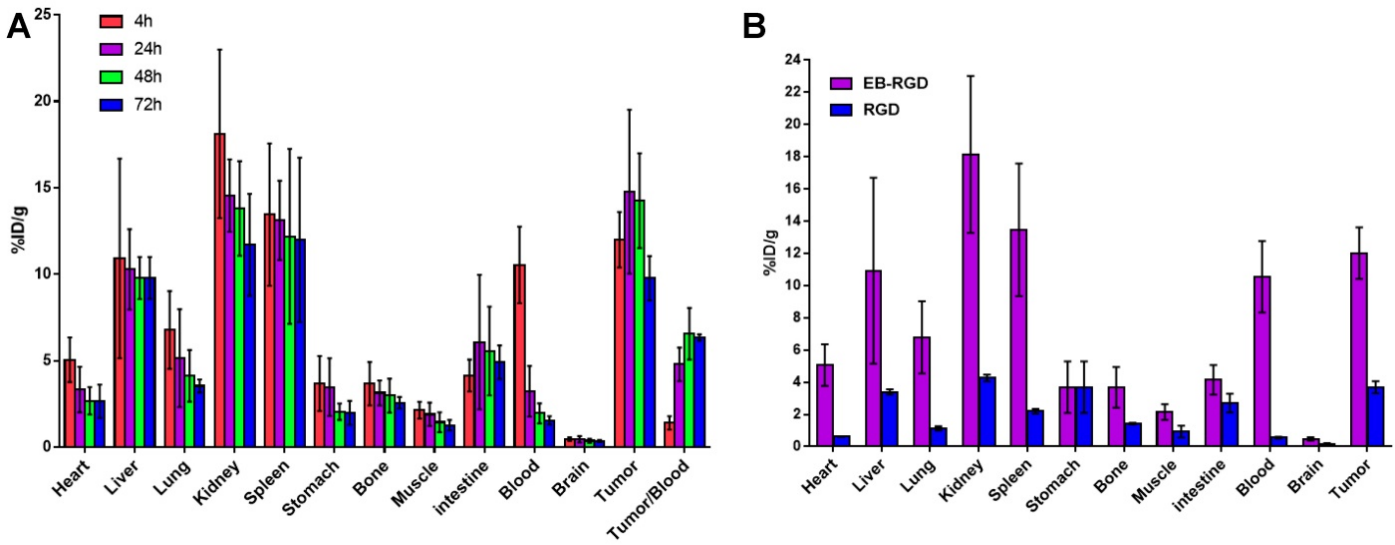
(A) Biodistribution of ^177^Lu-EB-RGD in MC38 tumor bearing mice at 4, 24, 48 and 72 h post-injection (n = 4/group). (B) Biodistribution comparison of ^177^Lu-EB-RGD and ^177^Lu-RGD in MC38 tumor bearing mice at 4 h post-injection (n = 4/group).

The biodistribution of ^177^Lu-EB-RGD in MC38 tumor-bearing mice was obtained through *ex vivo* counting in tissues collected from mice that were sacrificed at different p.i. time points (Figure 3A). The results obtained from these studies were generally consistent with previous observations from SPECT imaging. At 4 h p.i., ^177^Lu-EB-RGD was mainly accumulated in the blood (10.54 ± 2.21 %ID/g) and blood rich organs such as the liver (10.92 ± 5.77 %ID/g) and spleen (13.44 ± 4.11 %ID/g). Moreover, ^177^Lu-EB-RGD was cleared predominantly through the renal pathway, as evidenced by the high kidney uptake (18.13 ± 4.87 %ID/g). Tumor uptake at this time was 11.99 ± 1.60 %ID/g, and tumor-to-blood (T/B) ratio was 1.40 ± 0.39. By 24 h p.i., ^177^Lu-EB-RGD in the blood, liver, spleen, and kidneys had decreased to 3.23 ± 1.47, 10.29 ± 2.32, 13.11 ± 2.29, and 14.55 ± 2.09 %ID/g, respectively, whereas the tumor uptake and T/B ratio had increased to 14.78 ± 4.74 %ID/g and 4.79 ± 0.97, respectively. At 48 h p.i., ^177^Lu-EB-RGD tumor uptake had decreased slightly to 14.26 ± 2.73 %ID/g, while the uptake in blood (1.96 ± 0.58 %ID/g), liver (9.78 ± 1.21 %ID/g), spleen (12.19 ± 5.06 %ID/g), and kidneys (13.80 ± 2.73 %ID/g) had continued to decrease. Further clearance of ^177^Lu-EB-RGD from all organs was seen at 72 h p.i, with T/B ratio further increased to 6.35 ± 0.18. The biodistribution of ^177^Lu-RGD was also observed by way of comparison (Figure 3B).

Consistent with the SPECT findings, ^177^Lu-EB-RGD demonstrated significantly longer blood half-life than ^177^Lu-RGD, and the blood uptake of ^177^Lu-EB-RGD was nearly 18 times greater than that of ^177^Lu-RGD (10.54 ± 2.21 *vs.* 0.56 ± 0.06 %ID/g at 4 h p.i.). As a result, ^177^Lu-EB-RGD exhibited significantly higher tumor uptake than ^177^Lu-RGD (11.99 ± 1.60 *vs.* 3.69 ± 0.37 %ID/g 4 h p.i.).

### Combination of TRT and anti-PD-L1 antibody synergistically enhances anti-tumor efficacy in MC38 tumors

**Figure 4:**
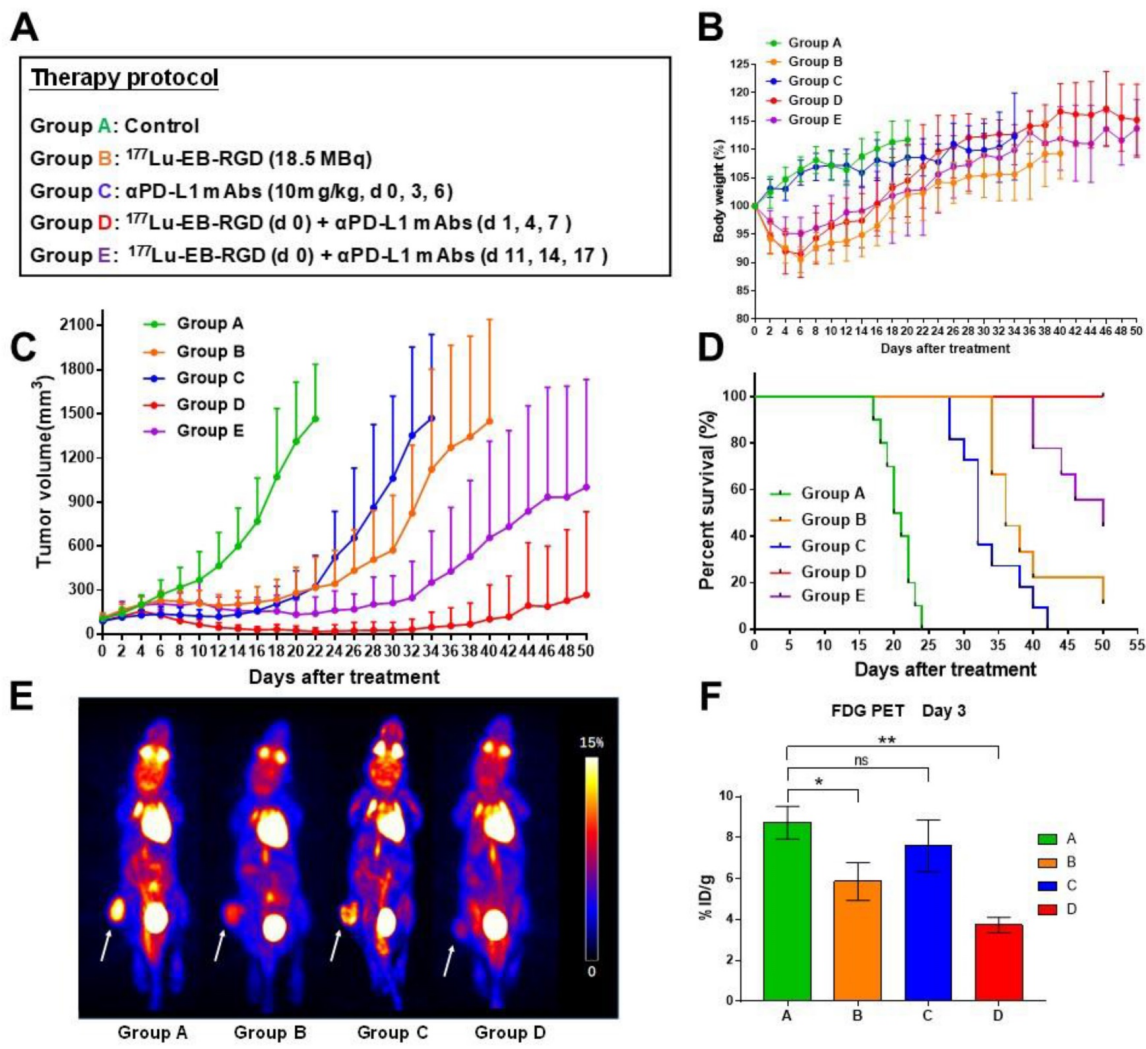
(A) Design of therapy protocol. (B) Body weight change. (C) Tumor volume. (D) Survival of mice (9-10/group) treated with saline (group A), ^177^Lu-EB-RGD (group B), αPD-L1 mAb (group C), concurrent combined therapy with ^177^Lu-EB-RGD and αPD-L1 mAb (group D) and sequential combined therapy with ^177^Lu-EB-RGD and αPD-L1 mAb (group E) at different days after treatment. (E) Projection PET images of MC38 tumor bearing mice injected with ^18^F-FDG 3 d after treatment with saline (group A), ^177^Lu-EB-RGD (group B), αPD-L1 mAb (group C) and combined therapy with ^177^Lu-EB-RGD and αPD-L1 mAb (group D). White arrows indicate the tumor location. (F) PET quantification of the images in Fig 4E. *P < 0.05, ** *P* < 0.01

To investigate whether combined therapy would be more efficacious than ^177^Lu-EB-RGD or anti-PD-L1 mAb alone, a study was performed with five groups (A-E), each including 9-10 MC38 tumor-bearing mice. The anti-tumor efficacy of each treatment was monitored over 2 months. The therapy protocols along with the relative tumor volumes and body weights of the mice from each group are shown in Figure 4A.

No systemic radiotherapy-related toxicity, as indicated by the animals' body weight, was observed (Figure 4B). Rapid tumor growth was observed in mice treated with saline (Group A), and all mice in this group had to be euthanized by day 24 due to excessive tumor volume (Fig 4C; green line). In mice treated with ^177^Lu-EB-RGD alone (Group B) and in those treated using anti-PD-L1 mAb alone (Group C), a significant delay in tumor growth was observed (Figure 4C; orange and blue lines). Starting from day 6, a significant difference in tumor volume was observed between the anti-PD-L1 mAb-treated and saline-treated groups (*P* = 0.007), while a significant difference between the ^177^Lu-EB-RGD and saline-treated group was observed from day 8 (*P* = 0.001). Mice in Groups B and C survived until day 39 and day 33 of the study, respectively, representing significantly greater survival than control mice (*P* < 0.0001 for both Groups B and C) (Figure 4.D). During treatment, a partial response (PR) was observed in two of nine mice treated with ^177^Lu-EB-RGD and in six of 10 mice treated with anti-PD-L1 mAb, but relapse was observed a few days later in both groups.

Next, enhancement of anti-PD-L1 immunotherapy by combination with ^177^Lu-EB-RGD was assessed. To evaluate the most appropriate timing of immunotherapy after radiotherapy, the anti-PD-L1 mAb was delivered in a concurrent or sequential manner with ^177^Lu TRT. The greatest therapeutic efficacy was observed in the concurrent combined therapy group (Group D), with further marked inhibition of tumor growth (Figure 4C; red line). Starting from day 8, significant differences in tumor volume were observed between Groups B and D (*P* = 0.013), while significant differences between Groups C and D were observed from day 10 after treatment (*P* = 0.034). These differences increased over time; most tumors had started to shrink by day 6 and had completely disappeared by day 26. During treatment, 2 of 10 mice showed PR, while 8 of 10 showed complete response (CR), with all of these maintaining CR for the duration of the study (until day 50). Therefore, mice that received concurrent combined therapy of ^177^Lu-EB-RGD and anti-PD-L1 mAb showed significantly better survival than those treated using ^177^Lu-EB-RGD alone (*P* < 0.0001) or anti-PD-L1 mAb alone (*P* < 0.0001) (Figure 4D). Moreover, 4 of the mice with complete tumor regression were rechallenged with 10^6^ MC38 cells on day 80, to further investigate whether immunologic memory was generated following treatment with TRT and anti-PD-L1 mAb. The results indicated that all 4 mice were able to completely reject rechallenged tumors.

In the group that received sequential combined therapy of ^177^Lu-EB-RGD and anti-PD-L1 mAb (Group E), a slightly less profound therapeutic efficacy was observed than in the group that received concurrent combined therapy (Group D), with the tumor volumes in Group E being slightly larger than those in Group D from day 8 after treatment (*P* = 0.007), but still significantly smaller than those in Groups B and C from day 16 until the end of the study (Figure 4C; purple line). CR was observed in 2 of 10 mice, while PR was observed in 4 of 10. Additionally, in Group E, survival was greater than that in Group B (*P* = 0.0007) and Group C (*P* < 0.0001), but not greater than that in Group D, wherein the mice survived until the end of the study (Figure 4D).

During treatment, we evaluated tumor metabolism using appropriate PET tracers. Specifically, ^18^F-FDG PET imaging, an indicator of cellular metabolism, was conducted 3 days after the initial treatment and showed significantly less ^18^F-FDG uptake in Groups B (treated with ^177^Lu-EB-RGD) and D (^177^Lu-EB-RGD radiotherapy concurrent with anti-PD-L1 immunotherapy) than that in the saline-treated group (Figure 4E). In addition, the difference between control and therapy group is smaller when the tumor-to-background ratio was used for quantification, but significant difference in Group B (8.32 ± 1.25 *vs.* 6.22 ± 0.92, *P* < 0.05) and Group D (8.32 ± 1.25 *vs.* 5.89 ± 0.92, *P* < 0.05) can still be observed. These results suggested reduced tumor metabolism in these mice. The mice treated using anti-PD-L1 mAb alone (Group C) also showed decreased FDG uptake compared to the control group, although this difference was not statistically significant (Figure 4F).

### Tumor biology after ^177^Lu targeted radionuclide therapy

**Figure 5:**
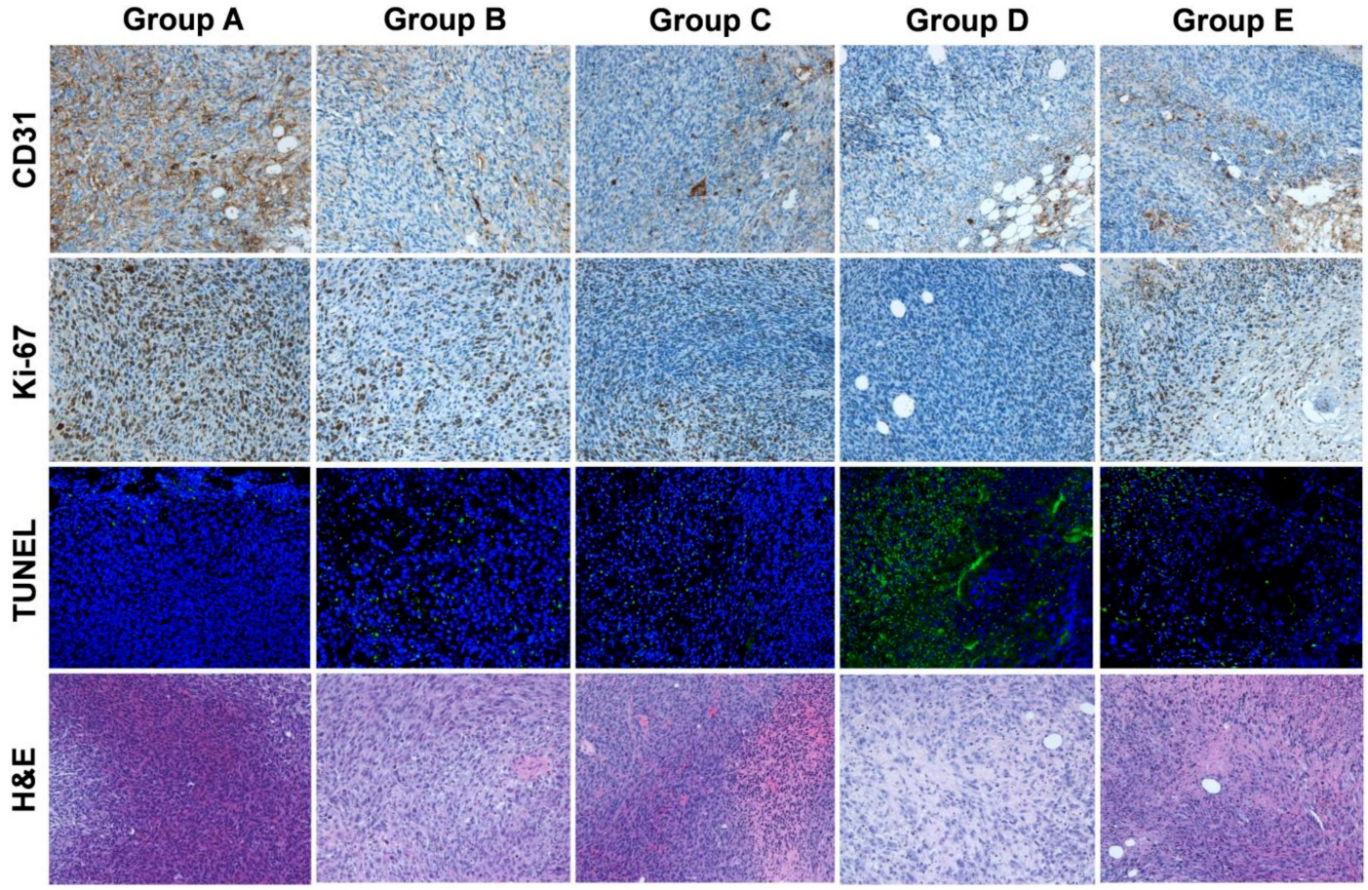
Immunofluorescence staining of excised tumors for CD31 (vascularity), Ki-67 (proliferation), TUNEL (apoptosis), and hematoxylin and eosin (H&E) after treatment with saline (group A), ^177^Lu-EB-RGD (group B), αPD-L1 mAb (group C), concurrent combined therapy with ^177^Lu-EB-RGD and αPD-L1 mAb (group D) and sequential combined therapy with ^177^Lu-EB-RGD and αPD-L1 mAb (group E). Tumor samples from groups A-D were collected at d 7 after treatment, and samples from group E were collected at d 14 after treatment (αPD-L1 mAb starting on d 11 of TRT).

CD31 staining to evaluate tumor vasculature, Ki-67 staining to evaluate tumor proliferation, and TUNEL staining to determine DNA damage were conducted on excised tumors in all five groups (A-E). In Groups D and E, which had large necrotic areas, we evaluated live cells on the tumor rim. As shown in Figure 5, the tumor vasculature in Group D (concurrent therapy using ^177^Lu-EB-RGD and anti-PD-L1 mAbs) had the lowest extent of all groups. Moreover, a relatively high percentage of cells stained positively for Ki-67 in Groups A, B, and C, while significantly reduced cell proliferation was observed in Groups D and E. Compared with the other four groups, Group D showed considerably more cell apoptosis, as depicted by TUNEL staining. Hematoxylin and eosin staining showed that most of the tumor area in Group D was already necrotic 7 days after the initial treatment, whereas tumors from the other four groups only had necrotic areas in the middle of the tumor, suggesting that the necrosis was due to insufficient blood supply and hypoxia.

### TRT up-regulates PD-L1 expression while TRT in combination with anti-PD-L1 antibody increases the infiltration of CD8+ T cells

**Figure 6:**
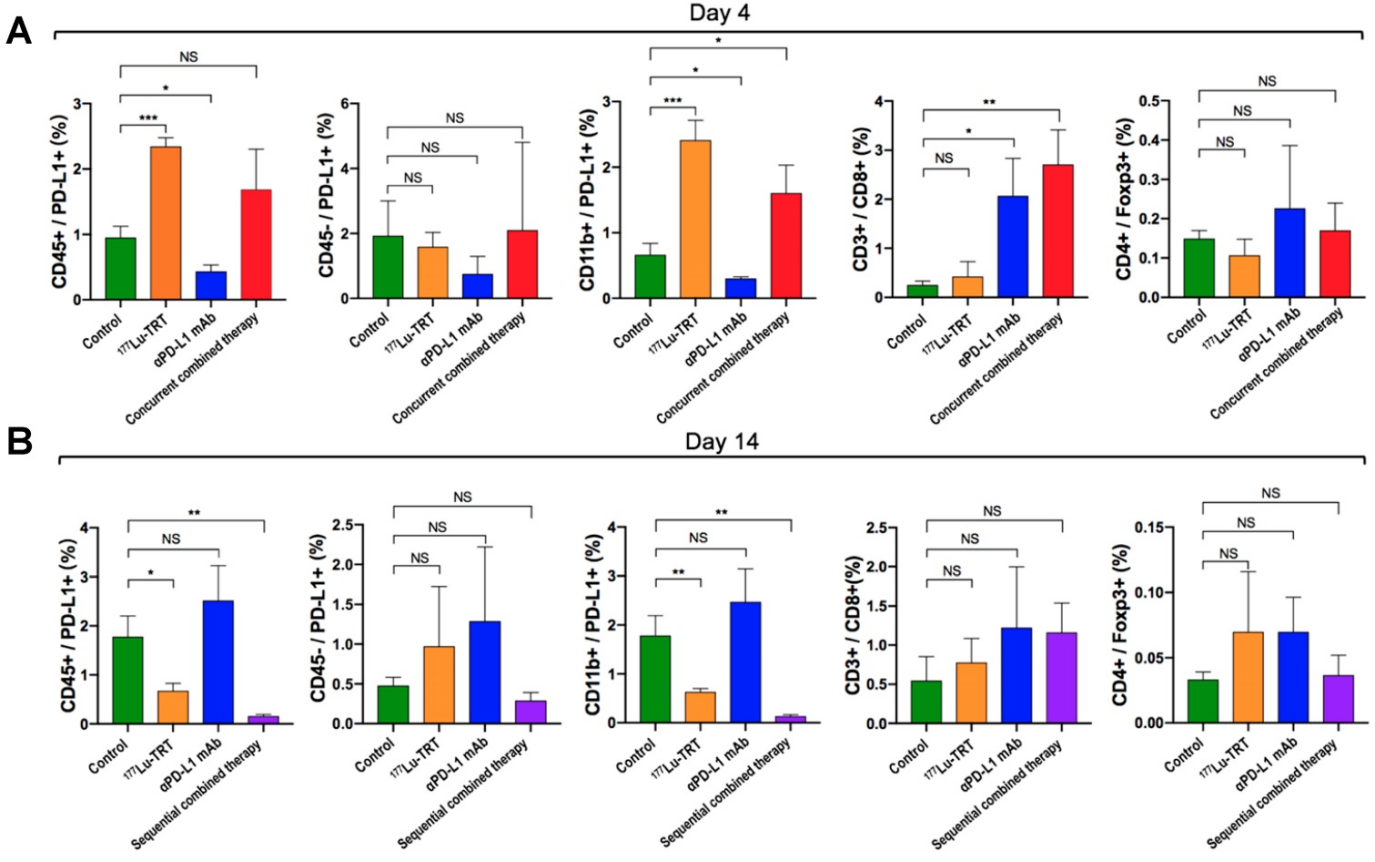
(A-B) Percentage of PD-L1^+^ immune cells (CD45^+^/PD-L1^+^, CD11b^+^/PD-L1^+^), PD-L1^+^ neoplastic cells (CD45^-^/PD-L1), CD8^+^ T cells and tumor-infiltrating regulatory T cells (CD4^+^/foxp3^+^) in MC38 tumors, which were measured by flow cytometry 4 d (A) and 14 d (B) after treatment with saline (green), ^177^Lu TRT (orange), αPD-L1 mAb (blue), concurrent combined therapy with ^177^Lu TRT and αPD-L1 mAb (red) or sequential combined therapy with ^177^Lu TRT and αPD-L1 mAb (purple) (n = 4/group). TRT = targeted radionuclide therapy. *P < 0.05, **P < 0.01, ***P<0.001.

To investigate alterations in the tumor microenvironment after TRT and immunotherapy and thus further elucidate the potential mechanisms of TRT combined with immunotherapy, PD-L1^+^ immune cells (CD45^+^/PD-L1, CD11b^+^/PD-L1), PD-L1+ neoplastic cells (CD45^-^/PD-L1), CD8^+^ T cells and tumor-infiltrating regulatory T cells (CD4^+^/Foxp3^+^) were assessed dynamically using both flow cytometry and multiplexed immunofluorescence. The flow cytometry analysis showed that, compared with the saline-treated control group (Group A), percentage of CD45^+^/PD-L1^+^ cells and CD11b^+^/PD-L1^+^ cells on day 4 were significantly increased in MC38 tumor-bearing mice treated using ^177^Lu TRT (Group B), whereas no significant difference was observed in the level of CD45^-^/PD-L1 and CD4^+^/ Foxp3^+^ cells between the control and therapeutic groups (Group B-D) (Figure 6A). However, on day 14 after the initial treatment, the levels of PD-L1-expressing CD45^+^ cells and CD11b^+^ cells in the group of ^177^Lu TRT (Group B) were significantly lower than those in the control group (Figure 6B). Moreover, MC38 tumors that had been subjected to concurrent therapy of TRT and anti-PD-L1 mAb (Group D) exhibited significantly more CD3^+^/CD8^+^ T cells than saline-injected controls, whereas this finding was not observed in mice treated using sequential therapy (Group E) (Figure 6B).

**Figure 7:**
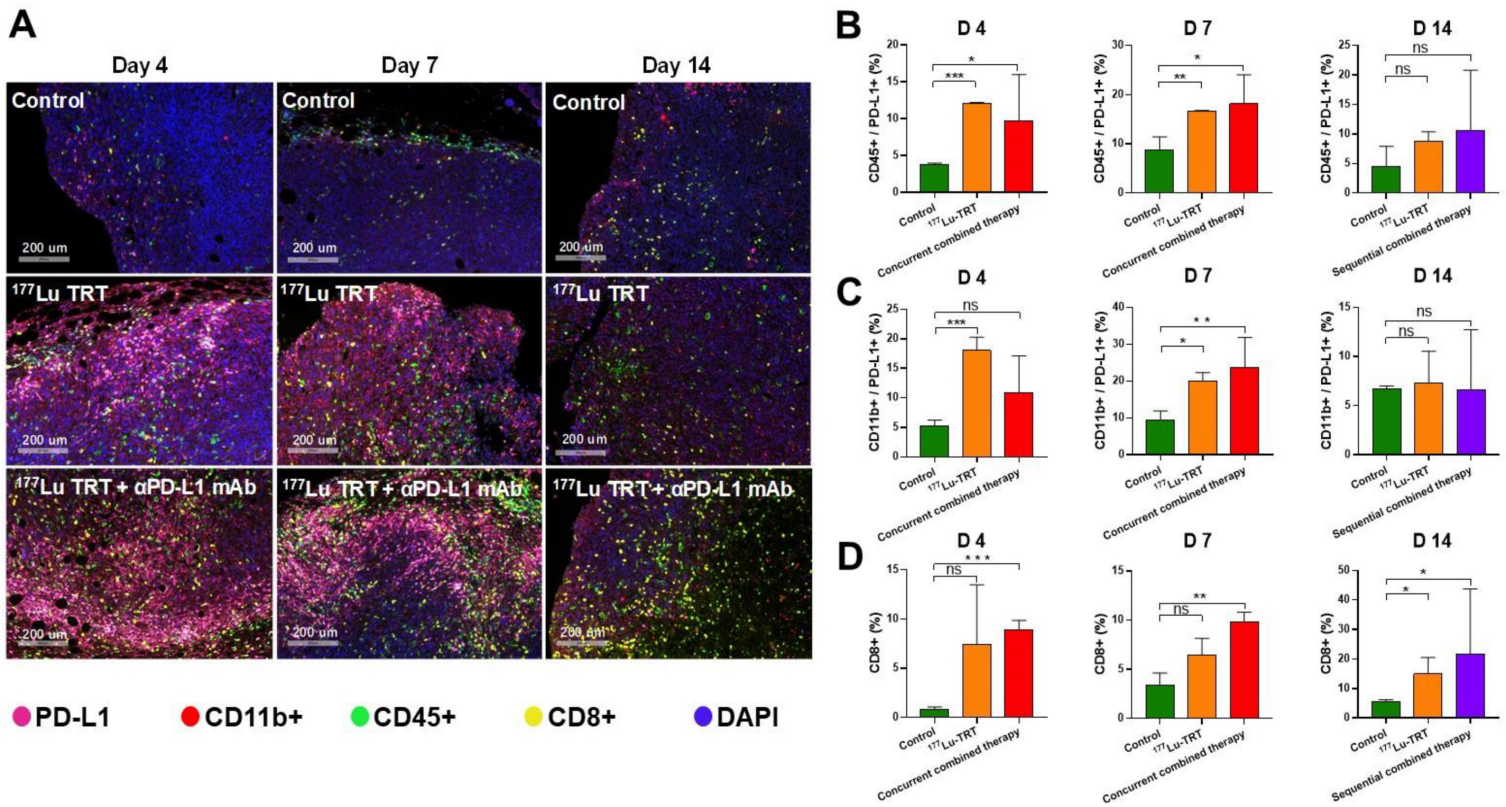
(A) Multiplexed immunofluorescence staining for PD-L1 (pink), CD11b+ (red), CD45+ (green), CD8+ (yellow) and DAPI (blue) in MC38 tumors, which were measured 4 d (left), 7 d (middle) and 14 d (right) after treatment with saline (control), ^177^Lu-EB-RGD targeted radionuclide therapy (TRT), concurrent combined therapy with ^177^Lu TRT and αPD-L1 mAb (day 4 and 7) or sequential combined therapy with ^177^Lu TRT and αPD-L1 mAb (day 14). Scale bar is 200μm (magnification, × 20). (B-D) Quantified results for multiplexed immunofluorescence staining presented in Figure 7A at different time points. *P < 0.05, **P < 0.01, ***P<0.001.

Multiplexed immunofluorescence analysis revealed similar results. Representative images of tumor section slides with multiplexed immunofluorescence staining are shown in Figure 7A. The levels of CD45^+^/PD-L1^+^ cells and CD11b^+^/PD-L1^+^ cells were significantly higher on days 4 and 7 in mice treated using ^177^Lu TRT than in saline-injected controls, whereas no significant difference was observed on day 14 (Figure 7 B-C). Moreover, the level of CD8^+^ T cells was significantly higher in mice treated using either concurrent (day 4 and 7) or sequential (day 14) combined therapy (Figure 7D). Taken together, these data indicate that TRT led to an acute increase in PD-L1 expression on T cells, and that TRT combined with anti-PD-L1 mAb synergistically enhances anti-tumor immunity by stimulating CD8^+^ T cell infiltration.

## Discussion

Blockade of the PD-1/PD-L1 axis has shown encouraging efficacy across multiple malignancies, with mAbs targeted against either PD-1 or PD-L1. Nonetheless, combination approaches may be required to improve response rates and generate durable anti-tumor immunity. There is substantial evidence to suggest that the tumor-specific immune response is subsequently triggered and enhanced during combinatorial treatment of radiotherapy and immunotherapy 31, 32. TRT is a branch of radiotherapy and flexibility is built into this system because TRT can be used to treat both localized and metastatic tumors through locoregional or systemic administration. Therefore, researchers must now elucidate how to best combine TRT with immunotherapy.

In the year of 2018, Jaeyeon et al. firstly reported the combination of TRT and immune checkpoint inhibitors (ICIs) in B16-F10 melanoma model 33. The results demonstrated that TRT in combination with ICIs showed significantly enhanced overall survival as compared with TRT or ICIs alone. However, the optimum timing between the 2 therapies and the underlie mechanism of combinatory therapy require further investigation. To address the limitation of this previous study, we explored the effect of PD-L1 blockade when combined with TRT in either concurrent or sequential administration in the present study. Consistent with the previous research, our data demonstrate that anti-PD-L1 mAb combined with ^177^Lu-EB-RGD, an integrin α_v_ß_3_-targeted TRT, improve anti-tumor efficacy and prolong overall survival compared with either treatment alone. The underlying mechanism is that TRT leads to acute inflammation and cell death which results in the migration of immune cells to the tumor site, thereby TRT can modulate the tumor microenvironment to activate immune response and thereafter enhance anti-tumor efficacy.

Previous studies have demonstrated that the PD-1/PD-L1 axis plays an essential role in host immune surveillance and in the regulation of the tumor microenvironment 32. Therefore, inhibition of this pathway may induce the immune response and generate long-lasting anti-tumor responses to radiotherapy. Indeed, in our present study, PD-L1 expression was up-regulated after delivery of TRT, and this elevated PD-L1 expression may act as a biomarker for predicting enhanced anti-tumor response by combination therapy of TRT and PD-1/PD-L1 mAbs. Moreover, the combination of anti-PD-L1 mAb with TRT can improve the effector phase of the immune response by activating stimulatory T cells in the tumor microenvironment. The cytotoxic CD8^+^ T cells are important T cells in the inhibition of tumor growth 13, while tumor-infiltrating regulatory T cells (Tregs) are key mediators of immune suppression that negatively regulate T-cell function and facilitate immune escape 34. Our data showed that the number of CD8^+^ T cells was significantly increased in mice exposed to combination therapy than in those given TRT or immunotherapy alone, whereas no increase was observed in the inhibitory immune cells Tregs, indicating that combined TRT and anti-PD-L1 mAb enhances host anti-tumor immunity and improves anti-tumor efficacy by stimulating CD8^+^ T cell infiltration, which alters the tumor microenvironment and results in a synergistic anti-tumor immunity. Furthermore, our data indicated that mice with complete tumor regression remained tumor free and were able to completely reject tumors following contralateral rechallenge. This suggests that TRT when combined with blockade of PD1/PD-L1 axis may generate protective immunologic memory in long-term survivors. Taken together, TRT seems to be enough to elicit a similarly heightened response when combined with PD-L1 blockade as previously seen with external beam therapy, even though the absorbed dose delivered/dose rate from ^177^Lu TRT is much lower when compared to external-beam radiotherapy.

Another major challenge is identifying the most beneficial time window for combining TRT and immunotherapy. In preclinical studies involving external-beam radiotherapy, immunotherapy has been administered either a few days before, concurrent with, or after radiotherapy. For example, in the study performed by Dewan *et al.* 13, delaying administration of anti-CTLA-4 mAb until 2 days after radiotherapy completion resulted in lower therapeutic efficacy than when the mAb was given 2 days before or on the day of radiotherapy completion, implying that immunotherapy should not be administered too late after radiotherapy. In another study by Dovedi *et al.* 35, an anti-PD-L1 mAb was administered on day 1 of the radiotherapy cycle (schedule A), day 5 of the cycle (schedule B), or 7 days after completion of radiotherapy (schedule C). The results showed that sequential treatment (schedule C) was ineffective for improving overall survival, and that schedules A and B were much more effective. Similar findings were observed in the present study, which showed that concurrent TRT and anti-PD-L1 mAb (administered on days 1, 4, and 7 after TRT) conferred significantly enhanced anti-tumor efficacy and longer overall survival than sequential therapy. Both Flow cytometry and multiplexed immunofluorescence analysis demonstrated that TRT can lead to an acute increase in PD-L1 expression on T cells at early time points (during days 4-7 after TRT), whereas no increase was observed at later time points, suggesting that sequential therapy with delayed blockade of PD-1/PD-L1 signaling axis on later time point of TRT may be less effective due to the disappearance of transient TRT-induced PD-L1 expression, thereby missing the optimal time window for anti-PD-L1 therapy. Although the detailed mechanism not yet has been clarified, the present study suggests that delivering TRT shortly before immunotherapy may be preferable, as the ICB mAbs may not be beneficial without the transient immune-activating effects of TRT.

Consistent with previous studies that use external-beam radiotherapy, the present study demonstrated that PD-L1 expression is up-regulated after TRT delivery, and that it remains elevated for at least 7 days after the initial treatment. Our data suggest that TRT can improve anti-tumor response, while the PD-1/PD-L1 axis can attenuate it. As such, early inhibition of this axis may be critical for generating a long-lasting, effective anti-tumor response. However, it is not yet clear how radiation is involved in the modulation of PD-L1 expression. One study by Gong *et al.* 32 implied that radiotherapy leads to up-regulation of PD-L1 expression on tumor cells through the activation of the P13K/AKT and STAT3 pathways, which suggested that radiation-induced up-regulation of PD-L1 expression is driven by activation of the oncogenic signaling pathway. In another study involving anaplastic lymphoma kinase-derived pulmonary adenocarcinoma, PD-L1 expression was up-regulated by activation of STAT3 and hypoxia-inducible factor-1α under both normoxic and hypoxic conditions 36. In addition, activation of the MEK/ERK signaling pathway is involved in PD-L1 modulation in NSCLC cells that harbor either EML4-ALK rearrangement or EGFR mutations 37. These findings reveal a direct link between oncogenic drivers and PD-L1 expression. However, to illustrate the detailed mechanisms of oncogenes that drive the expression of PD-L1 after radiation, further investigation is needed. It should be noted that our study also has some limitations. First of all, the whole study was only tested in MC38 tumor model, which is generally an anti-PD-1/PD-L1 sensitive and immunologically “hot” tumor model. Besides, the biological behavior and microenvironment of MC38 murine colon cancer is very different from human colon cancer, which usually does not respond to immunotherapy unless mismatch repair deficiency (dMMR). In addition, our present study mainly focused on the effects of combination therapy of ^177^Lu-labeled peptides and PD-L1 blockade, the mechanism behind could be further explored.

In summary, we have demonstrated that TRT can up-regulate PD-L1 expression, and that TRT combined with anti-PD-L1 mAb can stimulate CD8^+^ T cell infiltration, altering the tumor microenvironment and resulting in a synergistic anti-tumor immunity in MC38 colon carcinoma model. Moreover, our data revealed that the time window for this combination therapy may be critical to outcome, with concurrent rather than sequential therapy being more effective in terms of overall survival and long-term tumor control. This therapeutic combination may be a promising approach to treating metastatic tumors in which TRT can be used. Clinical translation of the result would suggest that concurrent rather than sequential blockade of the PD-1/PD-L1 axis combined with TRT improves overall survival and long-term tumor control.
